# Serum S100B: A Potential Biomarker for Suicidality in Adolescents?

**DOI:** 10.1371/journal.pone.0011089

**Published:** 2010-06-14

**Authors:** Tatiana Falcone, Vincent Fazio, Catherine Lee, Barry Simon, Kathleen Franco, Nicola Marchi, Damir Janigro

**Affiliations:** Cleveland Clinic-Lerner College of Medicine, Cleveland, Ohio, United States of America; Chiba University Center for Forensic Mental Health, Japan

## Abstract

**Background:**

Studies have shown that patients suffering from depression or schizophrenia often have immunological alterations that can be detected in the blood. Others reported a possible link between inflammation, a microgliosis and the blood-brain barrier (BBB) in suicidal patients. Serum S100B is a marker of BBB function commonly used to study cerebrovascular wall function.

**Methods:**

We measured levels of S100B in serum of 40 adolescents with acute psychosis, 24 adolescents with mood disorders and 20 healthy controls. Patients were diagnosed according to DSM-IV TR criteria. We evaluated suicidal ideation using the suicidality subscale of the Brief Psychiatric Rating Scale for Children (BPRS-C).

**Results:**

Serum S100B levels were significantly higher (p<0.05) and correlated to severity of suicidal ideation in patients with psychosis or mood disorders, independent of psychiatric diagnosis. Patients with a BPRS-C suicidality subscores of 1–4 (low suicidality) had mean serum S100B values +/− SEM of 0.152+/−0.020 ng/mL (n = 34) compared to those with BPRS-C suicidality subscores of 5–7 (high suicidality) with a mean of 0.354+/−0.044 ng/mL (n = 30). This difference was statistically significant (p<0.05).

**Conclusion:**

Our data support the use of S100B as an adjunctive biomarker to assess suicidal risk in patients with mood disorders or schizophrenia.

## Introduction

Every year, one million people die by suicide worldwide, and approximately every forty seconds there is a suicide attempt [1]. In fact, suicide is the fifth leading cause of death in patients between the ages of 5–14, and the third leading cause of death in young adults (15–24 years). In the last two decades, the rate of suicide in those aged 10–14 years has doubled. Up to 90% of the people who die by suicide have a diagnosable psychiatric disorder, such as a mood disorder, psychosis or substance abuse[1]. The most effective way to prevent suicide is through early recognition and treatment of these disorders [1].

Biomarker studies of suicidal patients have shown immunological abnormalities in patient with psychotic and mood disorders [2]–[7]. Immunological mechanisms have been described in the pathophysiology of schizophrenia and major depressive disorder, as evidenced by altered immunological measurements from a variety of blood, cerebrospinal fluid or brain samples [5], [8]–[12]. Microglial activation has been suggested in 4 postmortem studies in patients with schizophrenia [13]–[16]. Two postmortem studies have reported neuroinflammation as a potential contributor to increased suicidal behavior [17], [18]. Microgliosis in the anterior cingulated cortex, dorsolateral prefrontal cortex, hippocampus and mediodorsal thalamic nucleous has been reported in suicidal patients, independent of the diagnosis (schizophrenia vs. mood disorders), though it is difficult to elucidate if microglial activation is the cause or consequence of suicide [17], [18].

The role of stress is an important link between the psychosocial and neurobiological underpinnings of suicidal patients [2], [6], [11], [19]. Stress activates the inflammatory cascade, and as a result of this reaction microglia become activated [3], [17], [20]. In an effort to further elucidate the link between inflammation, neurological diseases and the blood-brain barrier, Bayard et al. reported BBB permeability alterations in 18% out of 90 suicide attempters [21].

Current methods to determine BBB breakdown are limited by cost (contrast-enhanced MRI) or invasiveness (lumbar puncture). Neither is suitable for broad-scale or frequent screening of populations at risk. Nevertheless, a surrogate marker of BBB function offers several advantages. These include the possibility of many determinations over time, testing under circumstances that are not suitable for MRI scans (emergencies, contraindications, or non-cooperative emotionally distressed patients), and possible screening in other selected cohorts (i.e., cancer). The development of alternative strategies to non-invasively monitored BBB function has been elusive, until the recent discovery of CSF and glial proteins present in plasma when the BBB is breached [22]–[25]. Our data in conjunction with other European and American studies have supported the hypothesis that S100B is associated with blood-brain barrier leakage. The negative predictive value of S100B for BBB leakage is comparable to contrast-enhanced MRI or iodine-contrast CT [22], [23], [25]–[29]. The goal of the present study was to test the hypothesis that suicidality in adolescents is accompanied by an increase in S100B values, possibly indicating S100B in the pathophysiology of suicidality.

## Methods

### Ethics Statement

This study was conducted with approval from the Cleveland Clinic IRB under IRB# 4406. All participants signed a written informed consent, as well as that of a parent or guardian if the subject was under the age of 18, and the research was done according to the principles expressed in the declaration of Helsinki. Subjects included 40 adolescents admitted to the Child and Adolescent Psychiatry Unit with psychosis and 24 with mood disorders (patients in the mood disorder group included some other externalizing diagnosis such us ADHD, ODD, IED, but the main reason for consulting psychiatry was the mood disorder). Inclusion criteria included: age between 12–18 years, and psychosis diagnosed by consensus of two child and adolescent psychiatrists using the DSM-IV TR criteria for first-episode psychosis: psychosis Not Otherwise Specified (N.O.S), schizophreniform disorder, schizoaffective disorder and schizophrenia diagnosed within the 6 months prior to admission. All patients with psychosis had at least one of the following symptoms described in their medical records: hallucinations, delusions or peculiar fantasies (fantasies that interfere with the perception of reality). Patient exclusion criteria included: psychosis secondary to a known medical condition, substance-induced psychosis, history of substance abuse, mild to severe mental retardation (IQ less than 70), autism and other chronic neurological disorders, and patients taking lithium.

We also included 20 healthy controls of comparable age and gender distribution after complete description of the research, written informed consent was obtained from the parents or legal guardians, and assent was obtained from the children. Sixty-four subjects were included in this study (25 non-psychotic and 39 psychotic); these were age and sex-matched (see Tables 1–4). The study group size was based on the meta-analysis by M.L. Schroeter and colleagues [30] comparing serum S100B values of clinically depressed subjects to age and gender matched controls. A power analysis of these data indicated that less than 25 total subjects are needed to study the significance between suicidality risk and serum S100B values. For these calculations, we assumed the variability in S100B levels in clinical depression and suicidality correlate perfectly. Details of this power analysis are provided in Supplemental Table S3.

**Table 1:** Gender, Age, BMI and S100B comparison of high and low risk of suicide.

Grouping	N	%Male	%Female	Age (Ave)	Age (SE)	BMI (kg/m^2^)_Ave_	BMI (SE)	S100β (ng/mL)_Ave_	S100β (SE)
Low Risk	34	50.0%	50.0%	14.50	0.54	25.74	1.89	0.152	0.020
**High Risk**	30	66.7%	33.3%	14.13	0.47	24.98	0.99	0.354	0.044
**Total:**	64								

**Table 2 pone-0011089-t002:** p Value Comparison for Non-Psychotic Subjects vs. Low Risk Group.

Grouping	N	Sex (EV)	Age (EV)	BMI (EV)	S100β (EV)
Low Risk	20	1.000	1.000	1.000	1.000
**High Risk**	5	0.328	0.930	0.292	**0.018**
**Total:**	25				

**Table 3 pone-0011089-t003:** p Value Comparison for Psychotic Subjects vs. Low Risk Group.

Grouping	N	Sex	Age (EV)	BMI (EV)	S100β (UV)
Low Risk	14	1.000	1.000	1.000	1.000
**High Risk**	25	0.212	0.504	0.305	**3.65E-05**
**Total:**	39				

**Table 4:** p Value Comparison for Psychotic + Non-Psychotic Subjects vs. Low Risk Group.

Grouping	N	Sex	Age (EV)	BMI (EV)	S100β (UV)
Low Risk	34	1.000	1.000	1.000	1.000
**High Risk**	30	0.183	0.616	0.732	**1.50E-04**
**Total:**	64				

(EV) = Equal Variance; (UV) =  Unequal Variance.

Two child and adolescent psychiatrists interviewed each patient and family or legal guardian to obtain a formal diagnosis and to assess the severity of symptoms. To assess suicidal behavior and possible risk factors, patient medical records were reviewed, including medical and psychiatric history, psychosocial assessments, all notes from hospitalizations, and past family psychiatric history. To obtain a score of 7 on the BPRS-C in this study, the patient had to have a suicide attempt within the week prior to admission, with evidence of bodily harm (n = 16). Subjects in both experimental groups were then administered the Brief Psychosis Rating Scale for Children (BPRS-C) [31] and the Positive and Negative Syndrome Scale (PANSS)[32] to measure severity of symptoms. Suicidality was assessed using the following anchored question for the BPRS-C: 1-no suicidality is present, 2-very mild (thoughts when angry), 3-mild (ocassional thoughts), 4-moderate (thoughts present in the last week), 5-moderately-severe (recurrent thoughts present almost daily), 6-severe (current suicidal plan), 7-extremely severe (patient attempted suicide within the last week). Information on medications taken during admission was collected from electronic medical charts to allow for inclusion in statistical analysis (see Tables 5 and 6). In this study we excluded patient with other diagnosis since suicidal attempts are increased in patients with mood disorders and schizophrenia.

**Table 5:** Medication comparison of psychotic, non-psychotic and combined groups sub-divided by suicidal ideation.

Grouping	#Meds Total (Ave)	# Meds SE	#Anti-Psychotic Meds (Ave)	#Anti-Psychotic Meds (SE)	#Anti-Dep Meds (Ave)	#Anti-Dep Meds (SE)
Low Risk (All):	1.68	0.26	0.56	0.10	0.47	0.09
**High Risk (All):**	1.87	0.21	0.83	0.13	0.40	0.10
Low Risk (Non Psych):	1.85	0.41	0.40	0.13	0.55	0.11
**High Risk (Non Psych):**	2.00	0.45	0.60	0.24	0.80	0.37
Low Risk (Psych):	1.43	0.25	0.79	0.11	0.36	0.13
**High Risk (Psych):**	1.84	0.24	0.88	0.15	0.32	0.10

**Table 6:** p Value Statistics.

Grouping	Meds Total	Anti-Psychotic Meds	Anti-Dep Meds
Low Risk (All):			
High Risk (All):	0.582	0.087	0.599
Low Risk (Non Psych):			
**High Risk (Non Psych):**	0.864	0.504	0.398
Low Risk (Psych):			
**High Risk (Psych):**	0.280	0.660	0.819

All patients samples underwent serum analysis of S100B protein. For S100B analysis, blood samples were collected and immediately centrifuged at 1,200×*g* for 10 min, and the supernatant serum was stored at −80°C. The S100B concentration was measured in all samples by the Sangtec 100 ELISA (Enzyme Linked Immunosorbent Assay) method (Diasorin, Stillwater, MN) using high and low level manufacturer provided controls to ensure proper assay performance. Serum collected in parallel to that for the S100B analysis was similarly stored at −80°C. The detection limit for the S100B assays was <0.01 ng/mL. Analyses of inflammatory cytokines were performed by multiplexed ELISA technique, by Quansys Biosciences (Logan, UT) as presented in Supplemental Table S2.

A student t-test was performed for age, gender and BMI and compared to that for S100B in both the non-psychotic and psychotic subject groups (as well as the total, combined grouping). Homogeneity of variance was objectively assessed using 5 different statistical methods (the O'Brien's test, the Brown-Forsythe test, Levene's test, Bartlett's test and the Welch ANOVA calculation) and the variance determined by the majority consensus. All statistical parameters were calculated using the SAS Institute Inc., software application JMP8®.

## Results

The subjects with psychosis and those with mood disorders were combined into a single set of 64 patients treated for psychiatric illness. They were then separated into two groups based on the BPRS-C subscale score for suicidality. Those with scores of 4 or lower were identified as “low risk” of suicide, while those scoring 5 or higher as “high risk”, we decided to use this definition of risk according to clinical experience and reports that patients with daily suicidal ideation have increase risk of suicide attempts[1].

**Figure 1:**
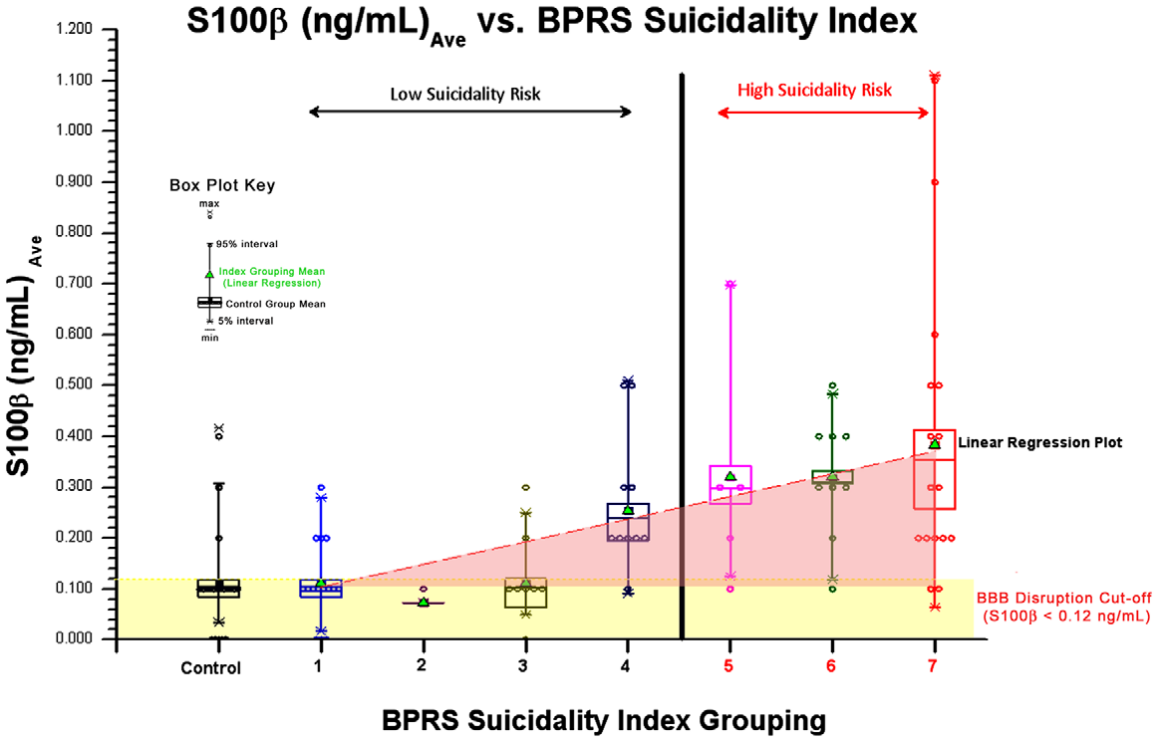
Correlation between S100B and suicidality. Measures of S100B in a group of 64 patients with psychosis and mood disorders, reveal a strong correlation between S100B and suicidality. Bivariate fit analysis of BPRS-C suicidal assessment (BPRS Suicidality Index) and S100B yielded a positive correlation with a corresponding p<0.01 and R^2^ correlation value of 0.273. Subjects with BPRS Index values <5 are identified as “low risk” of suicide, while those with values 5 or higher are identified as “high risk”. 20 control subjects are included for comparison. Patients with low suicidality risk are identified as having a BPRS suicidality index of 1–4 (black); high suicidality risk an index of 5–7 (red). Patient groupings are displayed as a box plot (see Box Plot key for description of max, min, group mean, and 5–95% intervals).

**Figure 2:**
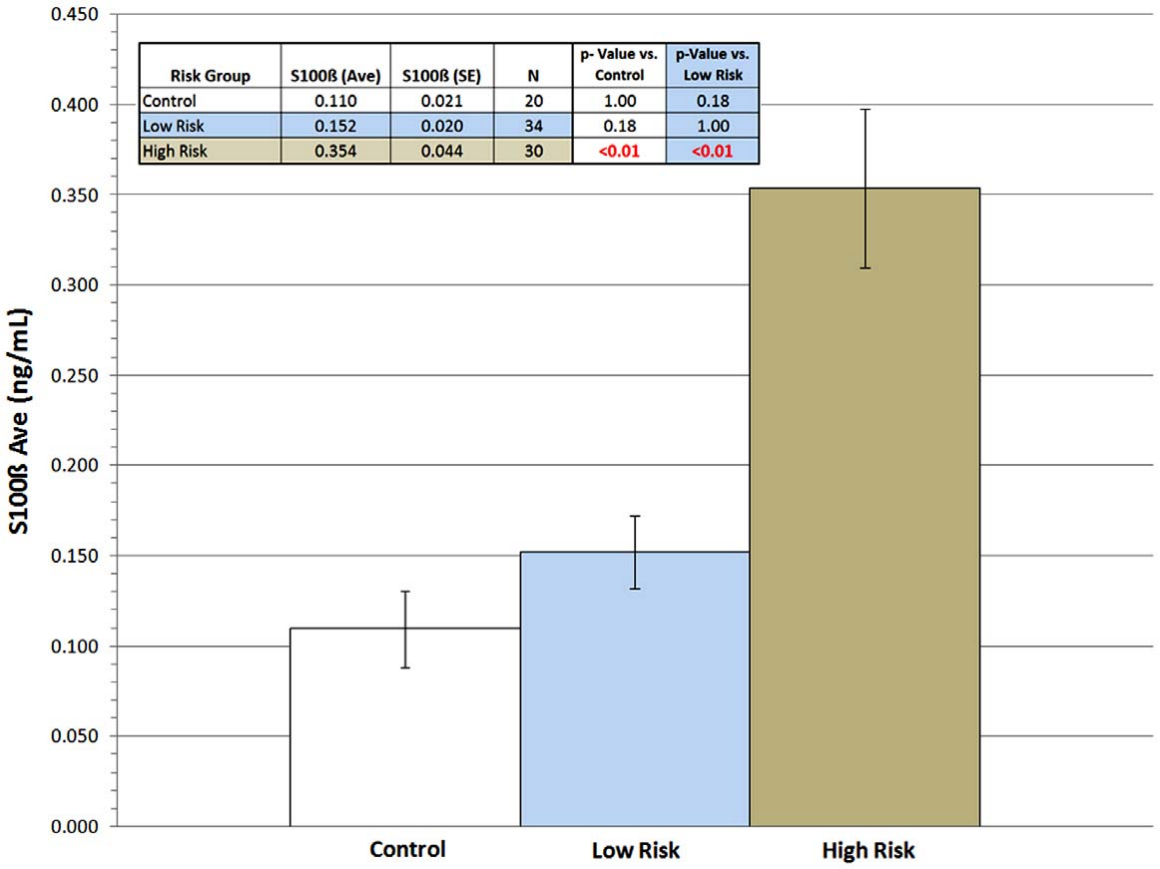
S100B comparison in low and high risk of suicide. In a group of 64 patients with either psychosis or mood disorders levels of S100B objectively predicted suicidal ideation (p<0.01). 20 control subjects are included for comparison.

Levels of S100B were found to be positively correlated (p<0.05) with suicidality, as measured by the BPRS-C suicidality subscore (see Figure 1). This was true for all subjects, regardless of diagnosis. When comparing the statistical difference between the “low risk” and “high risk” groups for suicide based on assessment of standard BPRS-C, the group with low risk had an average S100B levels of 0.152+/− SEM 0.020 and the group with high risk had an average S100B levels of 0.354+/−0.044, (see Figure 2). Difference between the groups was statistically significant (p<0.05, by Paired Student's t-test). This difference appeared to be unchanged by the exclusion of subjects having more than one psychotic episode. Bivariate fit analysis of BPRS-C suicidal assessment and S100B yielded a positive correlation with a corresponding p-value <0.01, and R^2^ correlation value of 0.273 (see Figure 1).

The study was controlled for age and gender based on the following data: Average age of patients with BPRS suicidality subscore of < or equal to 4 was 14.5+/−0.5 years (mean +/− SE), while those with subscore >4 (high suicide risk) were 14.1+/−0.5 years of age, with a p-value  = 0.616. The gender ratio of males to females in the low suicidality group was 17/17, while that in the high suicidality group was 20/10. While there appears to be a trend towards a predominance of males in the high suicidality risk group, the p-value does not show a significant difference between the two (p = 0.183). The study was also controlled for diagnosis of schizophrenia based on measurement of BPRS suicidality assessment. Those subjects with subscores < or equal to 4 were not significantly different for schizophrenic vs. non-schizophrenic subjects: Non-psychotic mean scores were 2.6+/−0.3 vs. 2.1+/−0.4 for psychotic subjects (p = 0.259). Likewise, those with subscores >4 were comparable with non-psychotic subjects averaging 6.2+/−0.4 and psychotic subjects 6.4+/−0.2 (p = 0.602). Similarly, no significant difference was observed in the height, weight or calculated body-mass index (BMI) when comparing the low and high suicidality risk groups (respective p-values of 0.248, 0.867 and 0.732). Additionally, BMI did not show any statistical significance (p<0.05) when comparing the low to high suicidality risk groups subdivided as either psychotic or non-psychotic (respective p-values of 0.31 and 0.29, see Tables 2–4).

In 2001, Rothermundt and colleagues had found that S100B levels were reduced significantly in a group of patients diagnosed with acute schizophrenia, where serum S100B was measured at baseline, in the absence of neuroleptic medication[33]. A subsequent paper by Hetzel et al., examined the effect on anti-depressant medication on serum S100B levels for a group of drug-free subjects diagnosed with major depression and 4 weeks post-medication treatment with either reboxetine or citalopram[34]. Although the S100B levels in these subjects were reduced after 4 weeks, the difference was not significantly significant (p = 0.28). Statistical analysis of the total number of medications, as well as anti-psychotic (neuroleptic), and anti-depressant medications separately was performed for high and low risk subjects who were identified as either psychotic or non-psychotic, as well as the two groups combined. No statistically significant difference was observed between the total, neuroleptic nor anti-depressant medication use between the low and high risk of suicidality for either psychotic or non-psychotic subjects or the two combined (see Supplemental data Table S1 and S4). To rule out traumatic injury possibly induced by attempted suicide, a comparison of 9 inflammatory cytokines (IL-1α,IL-1β, IL-2, IL-4, IL-6, IL-8, IL-10, IFN γ and TNFα) for subjects with high and low risk of suicidality (based on BPRS-C suicidality subscores) as well as for 17 healthy pediatric controls of comparable age and gender distribution is provided in Supplemental Table S2. Corresponding S100B serum levels were also measured. A statistically significant difference (p<0.05) was observed between the combined psychotic subjects (low and high risk of suicidality; n = 64) and pediatric controls for inflammatory cytokines IL-1 β and IL-8, as well as the protein biomarker, S100B.

## Discussion

The main finding of our study is that S100B is a biomarker that can help predict suicidality in adolescent patients with psychosis and mood disorders. This opens a new diagnostic venue for the psychiatrist and constitutes a new tool to study the pathobiology of suicide. The finding that S100B is a marker of psychiatric illness also expands our understanding on how blood-brain barrier disruption impacts brain function. Thus, in addition to seizure disorders [28], [35], [36], schizophrenia [37] and traumatic brain injury [38]–[40], pediatric psychosis and suicidality appear to be consequence, at least in part, of cerebrovascular malfunction. Our results also reiterate that S100B has no predictive value for any particular neurological disease, since elevated levels are present when the BBB is breached or when severe brain damage occurs. We also want to underscore that S100B is not a diagnostic tool for any particular neurological disease but rather an adjunctive means to predict evolution of disease or rule out the presence of underlying brain or cerebrovascular damage.

This was predicted by early studies by us and others showing that serum S100B elevation is not necessarily a consequence of neuronal cell death, or other pathological changes in the brain parenchyma [22], [24], [26]. The therapeutic and diagnostic value of S100B rather resides in its almost absolute negative predictive value [22]–[26], [36]–[41]. The negative predictive value of S100B for BBB leakage is comparable to contrast-enhanced MRI or iodine-contrast CT [22], [23], [25]–[29]. Thus, a normal S100B serum level in general does not warrant additional interventions or change of therapeutic course.

In recent years, there has been a proliferation of interest in the protein S100B, its many physiological roles and its behavior in various neuropathological conditions including mood disorders and schizophrenia [42], [43]. Suicide is a fatal consequence of a set of treatable diseases; based on our findings, S100B appears to be a reliable, easy accessible biomarker that can objectively help prevent suicide in this population. Suicide has a high prevalence in patients with mood disorders and schizophrenia.

A novel finding in this study is that S100B appears to be a marker of severity of risk of suicidality in patients with mood disorders and schizophrenia. This is in contrast to other studies where only cut-off values were indicative of presence or absence of disorder. The pathophysiological underpinnings of this are currently unknown but warrant further investigations.

The question remains, whether our results are in support of the link between S100B, blood-brain barrier disruption and inflammation. Studies by us or others have clearly shown that the etiological factors proceeding BBB disruption are indeed inflammatory in nature and comprise both cellular (monocytes) and molecular (IL1-β) factors [36], [44], [45]. Thus, it is possible that the originating factors leading to BBBD were indeed inflammatory. The triggers for these systemic changes are as of yet unknown, but have been shown to occur in other neurological diseases such as epilepsy[36], [46]. Similarly, we have found that two key inflammatory cytokines, IL-1β and IL-8, were elevated by a statistically significant level (p<0.05) in psychiatric patients (combined low and high risk subjects, based on BPRS-C suicidality subscores) compared to healthy control pediatric volunteers (see Supplemental Table S2).

To ensure that bodily harm, self induced by those patients attempting suicide, did not significantly contribute to any inflammatory processes which have been responsible for elevations in serum S100B an analysis of 9 inflammatory cytokines was undertaken in parallel to S100B measurements. Only BPRS-C suicidality subscores and S100B serum levels were statistically significantly different (p<0.05) when comparing the values of all measurements of low vs. high risk of suicidality patients (see Supplemental Table S2).

Recently, Hohoff and colleagues at the University of Muenster in Germany have identified genetic polymorphisms of the S100B gene in schizophrenic patients (specifically SNPs rs2186358, rs11542311, rs2300403 and rs9722) and have demonstrated that S100B protein and mRNA expressions are dependent on these gene polymorphisms[47]. This, however, seems unlikely given the facts that most of these identified polymorphisms are in a non-coding region of the gene and that no one has yet measured the frequency at which these polymorphisms occur. Although such polymorphisms may be proven as a key contributor to psychiatric illnesses or elevations in serum S100B, we feel these findings are premature to signify cause.

Though these results are promising, we realize that further prospective studies are needed to assess a broad clinical applicability of this biomarker for suicidality in different populations, ages, ethnicity, independent of the diagnosis. S100B may however become an important risk stratification tool for the assessment of suicidality in patients with severe mental disorders such as mood disorders and schizophrenia.

## Supporting Information

Table S1 Patient Demographic Summary: normalized by age, sex, and BMI for measuring the correlation between risk of suicidality and serum S100B levels.
(9.53 MB TIF)

Table S2 Statistical Comparison of S100B, BPRS Suicidality Index and 9 pro-inflammatory cytokine measurements for low and high risk of suicidal ideation and comparable age and gender distributed pediatric controls.
(6.60 MB TIF)

Table S3 Control vs. Depressed Patients Power Analysis for S100B based on the 2008 meta-analysis work by M.L. Schroeter and colleagues.
(5.02 MB TIF)

Table S4 Correlation Statistical Comparison of the measurements of depression, suicide and S100B outlined in Table S1.
(1.96 MB TIF)
