# Supplementary figures and images for: Serum S100B: A Potential Biomarker for Suicidality in Adolescents?
Source: PLoS One. 2010 Jun 14;5(6):e11089. doi: 10.1371/journal.pone.0011089 (PMC2885416; doi:10.1371/journal.pone.0011089)

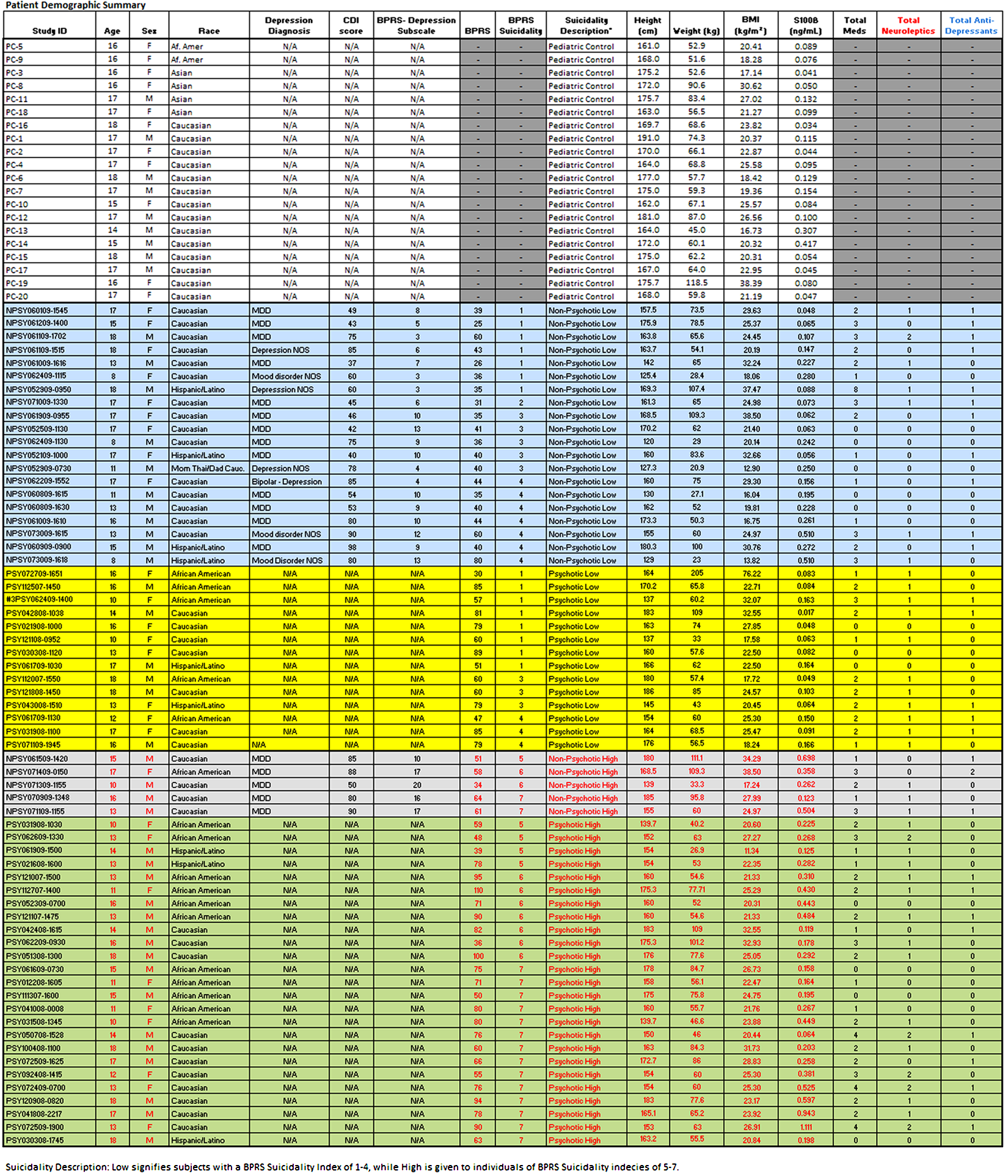

Supplement: Table S1 — Patient Demographic Summary: normalized by age, sex, and BMI for measuring the correlation between risk of suicidality and serum S100B levels. (9.53 MB TIF) [file pone.0011089.s001.tif]

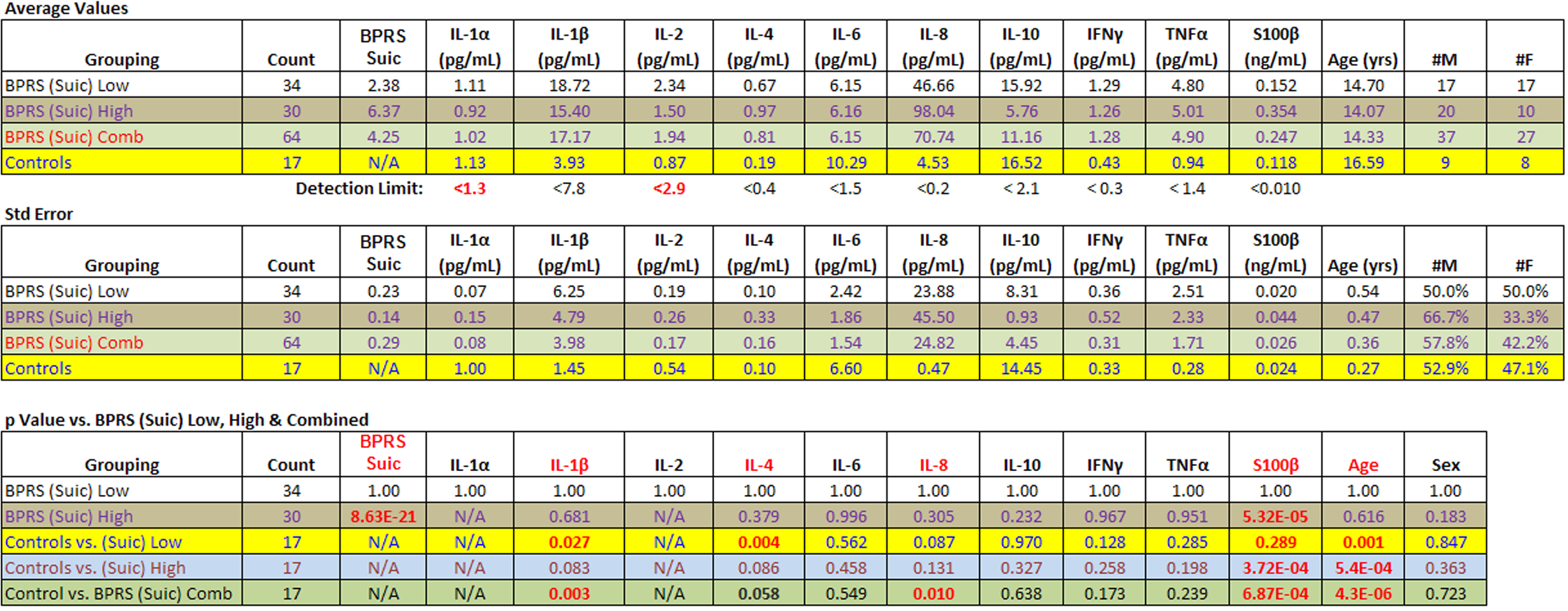

Supplement: Table S2 — Statistical Comparison of S100B, BPRS Suicidality Index and 9 pro-inflammatory cytokine measurements for low and high risk of suicidal ideation and comparable age and gender distributed pediatric controls. (6.60 MB TIF) [file pone.0011089.s002.tif]

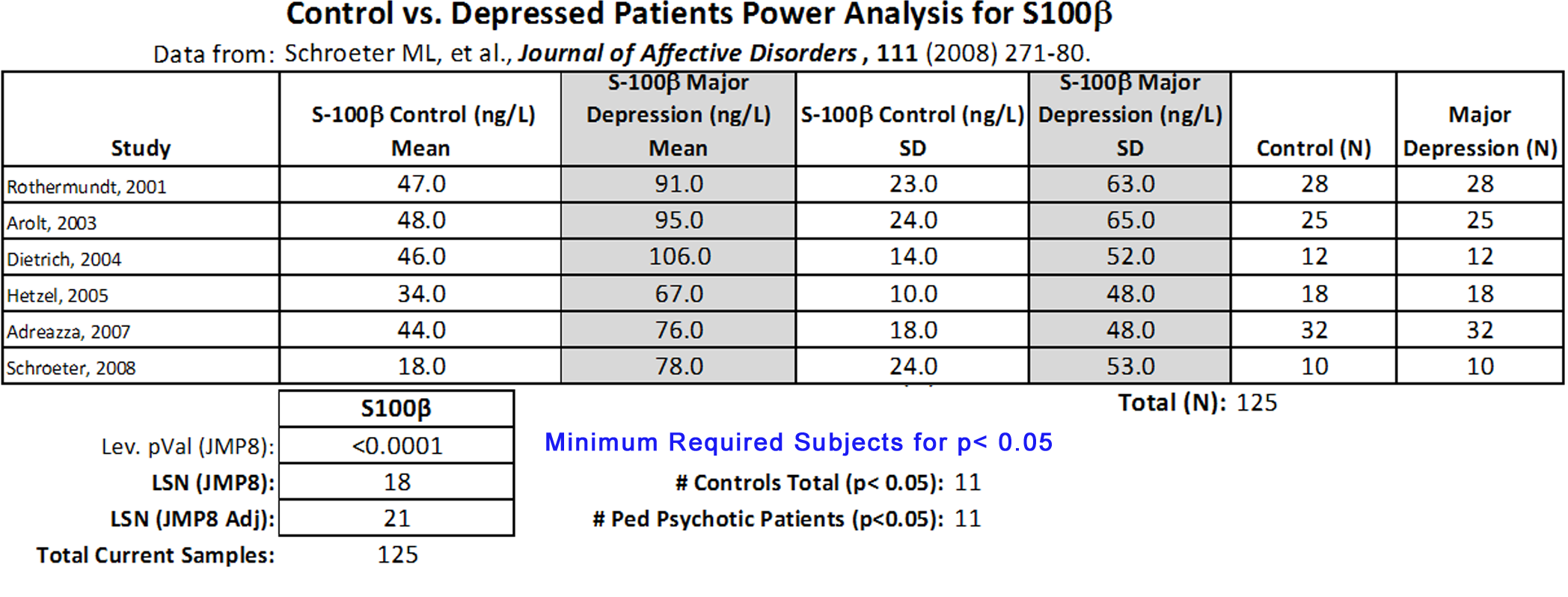

Supplement: Table S3 — Control vs. Depressed Patients Power Analysis for S100B based on the 2008 meta-analysis work by M.L. Schroeter and colleagues. (5.02 MB TIF) [file pone.0011089.s003.tif]

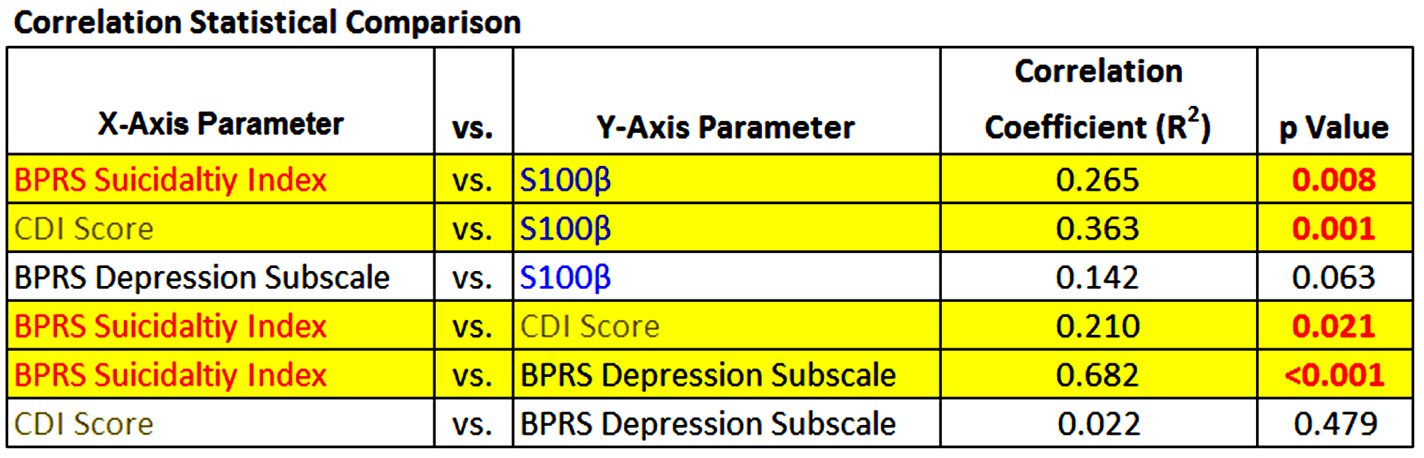

Supplement: Table S4 — Correlation Statistical Comparison of the measurements of depression, suicide and S100B outlined in Table S1. (1.96 MB TIF) [file pone.0011089.s004.tif]
